# Flexible Model Predictive Control for Bounded Gait Generation in Humanoid Robots

**DOI:** 10.3390/biomimetics10010030

**Published:** 2025-01-06

**Authors:** Tianbo Yang, Yuchuang Tong, Zhengtao Zhang

**Affiliations:** Institute of Automation, Chinese Academy of Sciences, Beijing 100089, China; yangtianbo2022@ia.ac.cn (T.Y.); yuchuang.tong@ia.ac.cn (Y.T.)

**Keywords:** gait generation, flexible C-T model, stable inversion, model predictive control

## Abstract

With advancements in bipedal locomotion for humanoid robots, a critical challenge lies in generating gaits that are bounded to ensure stable operation in complex environments. Traditional Model Predictive Control (MPC) methods based on Linear Inverted Pendulum (LIP) or Cart–Table (C-T) methods are straightforward and linear but inadequate for robots with flexible joints and linkages. To overcome this limitation, we propose a Flexible MPC (FMPC) framework that incorporates joint dynamics modeling and emphasizes bounded gait control to enable humanoid robots to achieve stable motion in various conditions. The FMPC is based on an enhanced flexible C-T model as the motion model, featuring an elastic layer and an auxiliary second center of mass (CoM) to simulate joint systems. The flexible C-T model’s inversion derivation allows it to be effectively transformed into the predictive equation for the FMPC, therefore enriching its flexible dynamic behavior representation. We further use the Zero Moment Point (ZMP) velocity as a control variable and integrate multiple constraints that emphasize CoM constraint, embed explicit bounded constraint, and integrate ZMP constraint, therefore enabling the control of model flexibility and enhancement of stability. Since all the above constraints are shown to be linear in the control variables, a quadratic programming (QP) problem is established that guarantees that the CoM trajectory is bounded. Lastly, simulations validate the effectiveness of the proposed method, emphasizing its capacity to generate bounded CoM/ZMP trajectories across diverse conditions, underscoring its potential to enhance gait control. In addition, the validation of the simulation of real robot motion on the robots CASBOT and Openloong, in turn, demonstrates the effectiveness and robustness of our approach.

## 1. Introduction

Humanoid robots are poised to transform productivity and daily life, yet a pivotal challenge remains in achieving stable operation in complex environments through adaptable, constrained gait generation [1,2].

Maintaining dynamic balance during walking requires the Zero Moment Point (ZMP) to remain within the robot’s support polygon [3,4]. However, due to the complex dynamics of humanoid robots, direct ZMP control is often suboptimal. Many approaches, therefore, simplify the robot model, linking ZMP variations to the center of mass (CoM) to achieve more effective control. The Linear Inverted Pendulum (LIP) model and Cart–Table (C-T) model are often utilized in this context, with their primary distinction being in how they treat ZMP—either as an input or output [5]. The LIP model is especially suited for inversion-based gait control [6,7,8]. By contrast, the C-T model, as the inverse of the LIP model, enables more intuitive ZMP tracking, such as preview control [9,10]. Consequently, ZMP trajectories generated by conventional methods do not fully account for real-world complexities [11].

To address these challenges, the approximation model needs to be improved to better approximate the inherent intrinsic properties of the robot. Numerous studies have introduced enhanced features to the LIP or C-T model [12,13,14,15]. Notably, ref. [16] developed an online running gait generator using a variable-height inverted pendulum model, while another study [17] integrated the LIP and linear pendulum (LP) models to achieve seamless transitions between single and double support phases. Additionally, ref. [18] introduced a simulated leg center of mass, effectively converting the gait generation problem of a multi-mass system into a single LIP-type problem. The flexible LIP model presented by [19,20] have demonstrated positive impacts on gait stability. Regardless of the model adopted, a robust control strategy is critical for real-time state control. Simplified model-based trajectory tracking control can effectively guide robotic motion [12,21], though it faces limitations with complex constraints.

Model predictive control (MPC) provides a versatile control method for constrained dynamic systems. It generates movements online from dynamic equations, enabling real-time adjustments of forthcoming motions based on potential conditions, efficiently solving a range of optimization problems globally in real time [22]. The research by [23] adapted the preview control method from [9] into an MPC framework, accounting for ZMP position constraints and using ZMP jerk as the solution variable to produce optimal outcomes and generate stable CoM trajectories. Ref. [24] proposed a robust method to meet constraints in low-level feedback control. Ref. [25] further enhanced robustness through MPC optimization adjustments. Ref. [26] integrated capture points into MPC, while ref. [27] developed a height-varying LIP-based MPC framework for robotic dynamic jumping. Additionally, ref. [28] based their MPC on a multi-mass model, supporting shorter prediction horizons and immediate response capabilities. Numerous MPC gait generation schemes have been implemented [29,30,31,32], satisfying constraints like ZMP balance, maximum step length [33,34], and LIP model instability [35,36,37,38], achieving favorable results. Most of these methods, however, rely on simplified LIP or C-T models [36,39,40,41]. While some research has extended to multi-mass models [18,28], they do not fully integrate dynamic equations into the MPC framework, thus lacking flexible modeling of the robot’s inherent characteristics.

To address the above challenges, this paper proposes an innovative Flexible MPC (FMPC) method that effectively approximates joint dynamics and integrates system flexibility, enabling humanoid robots to achieve stable, bounded motion in scenarios involving flexible joints and complex interactions. In contrast to existing MPC methods based on C-T models [23,41], the proposed flexible C-T model integrates the equivalent CoM of the robot with the second CoM through an elastic damping model to form a flexible C-T model and uses inverse derivation to obtain the flexible C-T model dynamical equations of the flexible C-T model, and dynamically extend it as a prediction model for FMPC, which enhances prediction flexibility and overcoming the limitations of conventional LIP or C-T models [7,9,23]. Furthermore, we integrate multiple constraints that emphasize CoM constraint, embed explicit bounded constraint, and integrate ZMP constraint, therefore substantially improving stability and dynamic performance for complex motion scenarios. Using ZMP velocity as the control input, the method formulates a standard Quadratic Programming (QP) problem. Through comparison, our proposed method shows clear advantages in meeting the demands of low prediction horizons and high-frequency responses. It also demonstrates strong stability under changes in CoM mass, speed, direction, and external disturbances. The proposed FMPC is anticipated to facilitate the realization of stable humanoid robot applications in complex environments across various fields. Thus, the key contributions of this research include:

1. This paper presents a Flexible MPC method that accurately models joint dynamics and integrates system flexibility, overcoming the challenge of generating flexible and stable gaits for humanoid robots, enabling bounded gait generation under varying conditions, therefore enhancing humanoid robots’ stable walking capability.
2. We employed an enhanced flexible C-T model as the predictive model, integrating the robot’s equivalent CoM with a spring-damping model and a secondary CoM to approximate the joint dynamics, therefore providing a more accurate representation of joint flexibility and an advantage in the flexibility of gait generation.
3. Through the integration of ZMP constraint, CoM distance constraint, and bounded constraint, we effectively constrained the uncertainties in the gait generation process, addressed the CoM trajectory divergence issue in gait generation, and achieved substantial improvements in FMPC stability and dynamic behavior representation.

The structure of the remaining sections is as follows: In Section 2, an overview of the FMPC framework is introduced. Section 3 focuses on the flexible C-T model and its state-space representation. Section 4 presents the FMPC-based gait generation method. In Section 5, we validate the proposed method through simulation experiments. The conclusion and future work are discussed in Section 6.

## 2. Overview of the Framework

In this section, we present the gait generation model of FMPC and discuss the overall framework of the proposed approach.

### 2.1. Description of the Problem

**Figure 1:**
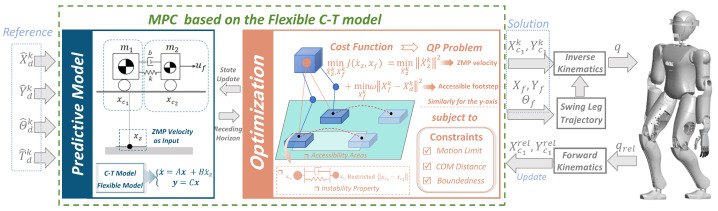
Block diagram of gait generation based on the FMPC framework.

The gait generation problem discussed in this paper focuses on generating the desired COM trajectories and foot positions based on a set of predefined gaits. As shown in Figure 1, this framework enables stable robot operation by generating flexible and bounded gaits.

We begin by defining the input gait sequence as: ${\hat{X}}_{d}^{k}={\left(x_{d}^{1}\ldots x_{d}^{F}\right)}^{T},{\hat{Y}}_{d}^{k}={\left(y_{d}^{1}\ldots y_{d}^{F}\right)}^{T}$, along with the corresponding foot orientation angle sequence and time interval sequence: ${\hat{\Theta }}_{d}^{k}={\left(\theta_{d}^{1}\ldots \theta_{d}^{F}\right)}^{T},{\hat{T}}_{d}^{k}={\left(T_{d}^{1}\ldots T_{d}^{F}\right)}^{T}$, where $T_{d}^{i}$ represents the time interval between the i+1 and *i* steps (useful for speed calculation), and ^^^ denotes predefined input variables.

In each MPC computation, the predefined sequence $\left({\hat{X}}_{d}^{k},{\hat{Y}}_{d}^{k}\right)$) of length *F* is refined into a sequence of footsteps $\left(X_{d}^{k},Y_{d}^{k}\right)$ of length $F^{\prime }$ that conforms to kinematic constraints. Within the prediction period *N* of the MPC, ${\hat{\Theta }}_{d}^{k}$ is kept constant. Please note that $F^{\prime }<F$, where the part of the sequence that does not reach *F* forms the tail of the sequence. The $X_{c_{1}}^{k},Y_{c_{1}}^{k}$ denotes the set of center-of-mass positions obtained from the MPC part, which is solved by the kinematics module to obtain the joint angles as the control inputs to the robot. At the same time, the actual CoM positions are computed by the kinematics module, which provides the feedback for the next MPC cycle.

Finally, footstep positions and orientations are interpolated to achieve end positions during the swing phase. Together with CoM positions, these undergo inverse kinematics to complete the movement plan.

### 2.2. FMPC Architecture

Our FMPC framework leverages predefined inputs and the state equations built from the flexible C-T model to solve an optimization problem in real time, yielding the actual walking footstep positions $\left(X_{f}^{k},Y_{f}^{k}\right)$ and the CoM positions $\left(X_{c_{1}}^{k},Y_{c_{1}}^{k}\right)$ within the robot’s control range.

The QP problem in this framework incorporates both footstep positions and ZMP velocity; the optimal ZMP velocity solution calculated by MPC is then applied in the model equation to determine CoM positions. We also add motion constraint equations, ensuring ZMP and kinematic feasibility. CoM distance constraints maintain system elastic stiffness, while boundedness constraints keep the CoM bounded relative to the ZMP, providing enhanced precision.

It is important to emphasize that this paper focuses primarily on the gait planning aspect, and the motion control details are not the central focus of this study.

## 3. The Flexible Cart–Table Model

This section introduces an enhanced flexible C-T model featuring an elastic layer and a secondary center of mass, which more accurately simulates the flexibility of the robot’s joints and driveline system.

### 3.1. Dynamical Model

**Figure 2:**
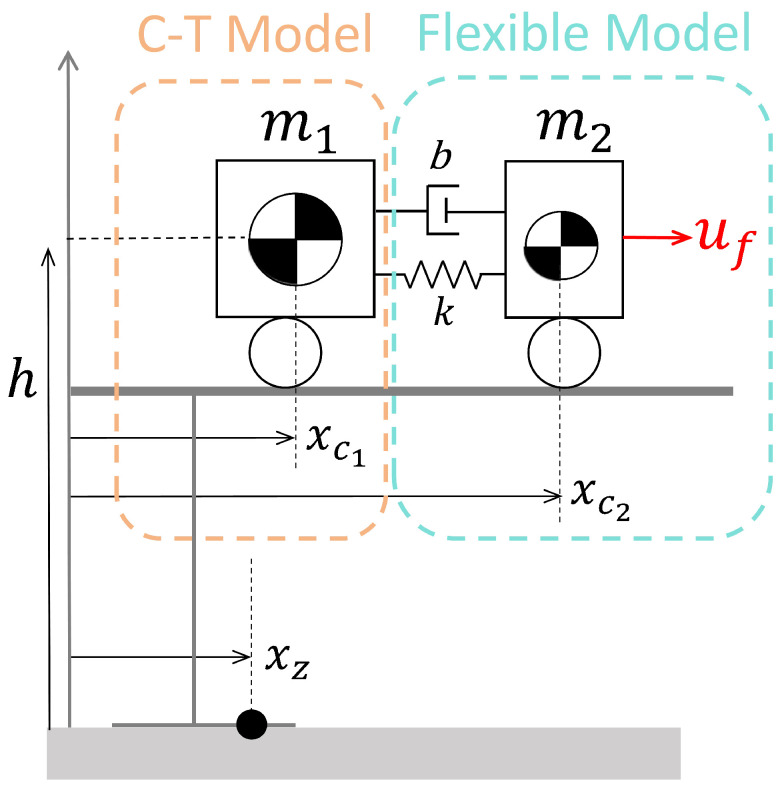
Flexible Cart–Table Model. The two carts are connected by a damper and a spring (constants *b* and *k*, respectively).

To model the interactions between the robot’s joints and its internal transmission system, we propose an enhanced flexible C-T model that incorporates spring-damping elements and a second center of mass (CoM) to improve its flexibility. The extended flexible C-T model is illustrated in Figure 2.

To simulate the robot’s internal elastic components, we represent the system as two interconnected carts. The dynamic equations governing the motion of the two carts $m_{1}$ and $m_{2}$ are given as follows:

(1) $$\begin{array}{c} m_{1}{\ddot{x}}_{c_{1}}=-k\left(x_{c_{1}}-x_{c_{2}}\right)-b\left({\dot{x}}_{c_{1}}-{\dot{x}}_{c_{2}}\right) \end{array}$$

(2) $$\begin{array}{c} m_{2}{\ddot{x}}_{c_{2}}=k\left(x_{c_{1}}-x_{c_{2}}\right)+b\left({\dot{x}}_{c_{1}}-{\dot{x}}_{c_{2}}\right)+u_{f} \end{array}$$

where $m_{1}$ and $m_{2}$ are located at positions $x_{c_{1}}$ and $x_{c_{2}}$, respectively, with $m_{2}\ll m_{1}$. The carts are connected by a spring-damper mechanism, with spring constant *k* and damping coefficient *b*. Control input is applied to cart $m_{2}$, allowing active acceleration; cart $m_{1}$ is accelerated solely by the spring-damper force, with its acceleration matching that of an individual cart. Thus, cart $m_{2}$ represents all dynamics preceding the robot’s internal elastic components, and cart $m_{1}$ represents everything following them.

Although two carts with mass are set in the dynamic model, for simplification, we assume cart $m_{1}$ as the dominant factor and exclude cart $m_{2}$ from the ZMP calculation. Therefore, we can derive the ZMP equation as follows:

(3) $$\begin{array}{c} {\ddot{x}}_{c_{1}}=\eta^{2}\left(x_{c_{1}}-x_{z}\right) \end{array}$$

where $\eta^{2}=g/h$. By performing a Laplace transform, we can derive the transfer function between ${\ddot{x}}_{c_{1}}$ and $x_{z}$, which corresponds to the basic C-T model. Hence, this part is regarded as the C-T model $F_{ct}\left(s\right)$ within the system.

(4) $$\begin{array}{c} F_{ct}\left(s\right)=\frac{X_{z}\left(s\right)}{{\ddot{X}}_{c_{1}}\left(s\right)}=\frac{1-\frac{1}{\eta^{2}}s^{2}}{s^{2}} \end{array}$$

Applying the Laplace transforms of (1) and (2) and performing some calculations and simplifications lead to the transfer function between $u_{f}$ and $x_{c_{1}}$, which takes the following form:

(5) $$\begin{array}{c} F_{flex}\left(s\right)=\frac{{\ddot{X}}_{c_{1}}\left(s\right)}{U_{f}\left(s\right)}=\frac{bs+k}{m_{1}m_{2}s^{2}+\left(m_{1}+m_{2}\right)\left(bs+k\right)} \end{array}$$

As observed in (5), the system possesses a stable zero at −k/b, which confirms that the system is stable and also minimum-phase. Thus, it can be regarded as the flexible model $F_{flex}\left(s\right)$ within the overall system.

**Figure 3:**
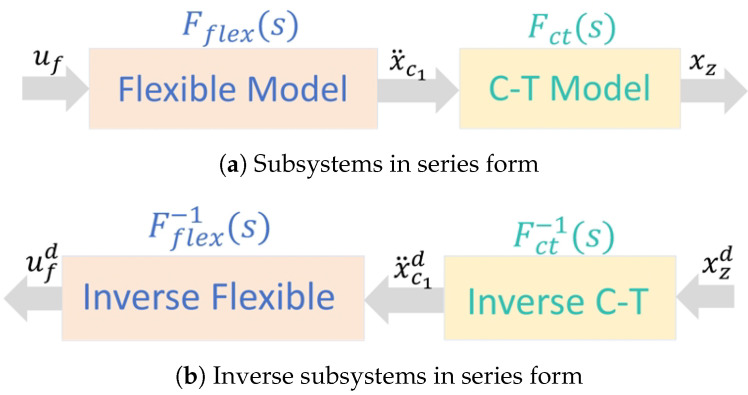
Reciprocal representation of series systems.

Accordingly, we can deduce that the entire system can be decomposed into two subsystems connected in series, as illustrated in Figure 3a. The input $u_{f}$ goes through the flexible system, resulting in the intermediate variable ${\ddot{x}}_{c_{1}}$, which then produces the output $x_{z}$ through the C-T model.

**Remark** **1.**  *Conventional LIP or C-T models [9] simplify the robot’s total mass as a single point mass, often missing the dynamic interplay between joints. The flexible C-T model integrates spring-damper elements and an additional CoM link, closely approximating the robot’s internal transmission dynamics, including motors, gears, lightweight links, and potential proportional-derivative (PD) controllers, which can be modeled as springs and dampers. Despite these enhancements, it retains linearity, making it readily applicable.*

### 3.2. Stable Inversion for the Flexible C-T Model

Due to the presence of a non-minimum-phase system $F_{ct}\left(s\right)$, directly controlling the ZMP can result in instability. To prevent this, the inversion method [7] can be used to derive bounded CoM trajectories from the desired ZMP trajectory, ensuring the internal stability of the system:

(6) $$\begin{array}{c} F_{ct}^{-1}\left(s\right)=\frac{s^{2}}{1-\frac{1}{\eta^{2}}s^{2}} \end{array}$$

In the C-T model (6), the cart’s movement, which corresponds to the CoM trajectory, allows us to calculate the resulting ZMP trajectory $x_{z}$ using the ZMP Equation (3). However, gait generation is the inverse problem, where the system’s motion should be computed from a given ZMP trajectory $x_{z}^{d}$.

Then, for the flexible CT model, we aim to find bounded trajectories for $x_{c_{1}}$. The preceding discussion decomposed the flexible C-T Model into two subsystems connected in series. Therefore, the inversion of the flexible C-T Model can be seen as the series connection of two inverse systems, where the output $x_{z}$ is replaced by input $x_{z}^{d}$.

As noted earlier, since the flexible model is a minimum-phase system, its inverse system $F_{flex}^{-1}\left(s\right)$ is also stable. The constraints related to boundedness only pertain to the unstable parts of the system, specifically addressing the unstable components of the LIP model. Therefore, the flexible C-T model can be transformed into a serial model as illustrated in Figure 3b.

To explicitly represent the inverse of the flexible model system, we use the input ${\ddot{x}}_{c_{1}}^{d}$ of the inverse system to express the output $u_{f}^{d}$. Using the known Equations (1) and (2), and performing differentiation, we obtain the following equation:

(7) $$\begin{array}{cc} {\dddot{x}}_{c_{1}} & =\frac{k}{m_{1}}\left({\dot{x}}_{c_{2}}-{\dot{x}}_{c_{1}}\right)+\frac{b}{m_{1}}\left({\ddot{x}}_{c_{2}}-{\ddot{x}}_{c_{1}}\right)\\ \; & =\left(\frac{k}{b}-b\tilde{M}\right){\ddot{x}}_{c_{1}}-\frac{k^{2}}{bm_{1}}\left(x_{c_{2}}-x_{c_{1}}\right)+\frac{bu_{f}}{m_{1}m_{2}} \end{array}$$

with

$$\begin{array}{c} \tilde{M}=\frac{1}{m_{1}}+\frac{1}{m_{2}}=\frac{m_{1}+m_{2}}{m_{1}m_{2}} \end{array}$$

This allows us to obtain the expression for the output $u_{f}^{d}$ of the inverse flexible model system. In applying the inverse flexible model system, the bounded CoM ${\ddot{x}}_{c_{1}}^{d}$ output from the LIP model is used as input to derive the output, which remains bounded for any state. Furthermore, it should be noted that the output also depends on the third derivative of $x_{c_{1}}$.

### 3.3. Approximation of High-Frequency Zeros

In the system, the controller frequently handles higher-order derivatives of state variables, like pulse inputs of the ZMP, which may become unstable due to numerical integration errors. To improve stability and decrease computational complexity, we use a high-frequency zero approximation method to simplify the control process.

We assume $F_{flex}^{0}\left(s\right)$ represents the flexible model system with added high-frequency zeros:

(8) $$\begin{array}{c} F_{flex}^{0}\left(s\right)=\left(1+\tau s\right)F_{flex}\left(s\right) \end{array}$$

This approach is essential for enhancing the control system’s response to high-frequency disturbances, which are especially prevalent in the operation of humanoid robots in dynamic environments. By incorporating high-frequency zeros into the system’s transfer function, we can efficiently suppress unwanted oscillations and greatly improve the system’s robustness to external perturbations.

To more intuitively derive the flexible model system, we describe it by formulating its state equations. We first assume the control variables of the flexible model as $x_{f}={\left(x_{c_{1}},{\dot{x}}_{c_{1}},x_{c_{2}},{\dot{x}}_{c_{2}}\right)}^{T}$, and the output as $y_{f}={\ddot{x}}_{c_{2}}$. Based on (1) and (2), we obtain:

(9) $$\begin{array}{cc} {\dot{x}}_{f} & =A_{f}x_{f}+B_{f}u_{f}\\ y_{f} & =C_{f}x_{f} \end{array}$$

where

$$\begin{array}{cc} A_{f} & =\left(\begin{array}{cccc} 0 & 1 & 0 & 0\\ -\frac{k}{m_{1}} & -\frac{b}{m_{1}} & \frac{k}{m_{1}} & \frac{b}{m_{1}}\\ 0 & 0 & 0 & 1\\ \frac{k}{m_{2}} & \frac{b}{m_{2}} & -\frac{k}{m_{2}} & -\frac{b}{m_{2}} \end{array}\right),B_{f}=\left(\begin{array}{c} 0\\ 0\\ 0\\ \frac{1}{m_{2}} \end{array}\right)\\ C_{f} & =\left(\begin{array}{cccc} -\frac{k}{m_{1}} & -\frac{b}{m_{1}} & \frac{k}{m_{1}} & \frac{b}{m_{1}} \end{array}\right) \end{array}$$

Simplifying (8) yields an expression similar to (5), with α and β representing the integrated results of the parameter calculations.

(10) $$\begin{array}{c} F_{flex}^{0}\left(s\right)=\frac{\alpha s+\beta }{m_{1}m_{2}s^{2}+\left(m_{1}+m_{2}\right)\left(bs+k\right)}+\frac{b\tau }{m_{1}m_{2}} \end{array}$$

By comparing (5), we notice an additional constant term. According to the system’s transfer function equation $G\left(s\right)=C{\left(sI-A\right)}^{-1}B+D$, the extra constant term in the flexible model system with added high-frequency zeros corresponds to the input matrix in the output equation of the $F_{flex}^{0}$ system, denoted as $D_{0}$. Additionally, we find that in the non-constant portion of the $F_{flex}^{0}$ system, only the numerator coefficient has changed. Likewise, the state matrix in the output equation also changes accordingly, denoted as $C_{0}$. Thus, we obtain the output equation for the system with added high-frequency zeros as:

(11) $$\begin{array}{c} {\ddot{x}}_{c_{1}}=C_{0}x_{f}+D_{0}u_{f} \end{array}$$

where

$$\begin{array}{c} C_{0}=\left(\begin{array}{cccc} -\frac{\beta }{m_{1}} & -\frac{\alpha }{m_{1}} & \frac{\beta }{m_{1}} & \frac{\alpha }{m_{1}} \end{array}\right),D_{0}=\frac{b\tau }{m_{1}m_{2}} \end{array}$$

Thus, the output of the inverse flexible model system can be expressed as:

(12) $$\begin{array}{c} u_{f}=-D_{0}^{-1}C_{0}x_{f}+D_{0}^{-1}{\ddot{x}}_{c_{1}} \end{array}$$

The output avoids the impact of the third derivative of $x_{c_{1}}$ as compared to (7), thus eliminating the instability caused by higher-order derivative integration errors.

**Remark** **2.**  *Through a detailed derivation of the flexible C-T model system, we obtain the complete dynamic equations, which maintain the stability of the system while adding an extra second CoM and spring-damping compared to the conventional C-T model. The analysis of the system shows the potential of the flexible C-T model in dealing with complex situations.*

## 4. Gait Generation Based on Flexible MPC

In this section, we incorporate the flexible C-T model into the prediction equations of the FMPC, constructing a comprehensive FMPC framework that integrates multiple constraints, including the ZMP constraint, CoM distance constraint, and boundedness constraint.

### 4.1. Motion Model

Considering the characteristics of the flexible C-T model, the x and y components of CoM and ZMP motions can be considered decoupled. In the FMPC framework presented in this paper, the control variable is the ZMP velocity $\dot{x_{z}}$ in order to obtain a smoother trajectory. In particular, this paper will consider a fifth-order system for the dynamic expansion of the flexible C-T model:

(13) $$\begin{array}{c} \dot{x}=Ax+B{\dot{x}}_{z} \end{array}$$

where

$$\begin{array}{c} A=\left(\begin{array}{ccccc} 0 & 1 & 0 & 0 & 0\\ 0 & 0 & 0 & 1 & 0\\ -\frac{k}{m_{1}} & \frac{k}{m_{1}} & -\frac{b}{m_{1}} & -\frac{b}{m_{1}} & 0\\ a_{41} & a_{42} & a_{43} & a_{44} & a_{45}\\ 0 & 0 & 0 & 0 & 0 \end{array}\right),B=\left(\begin{array}{c} 0\\ 0\\ 0\\ 0\\ 1 \end{array}\right) \end{array}$$

We take CoM positions of the two carts and the ZMP position as control variables: $x={\left(x_{c_{1}},x_{c_{2}},{\dot{x}}_{c_{1}},{\dot{x}}_{c_{2}},x_{z}\right)}^{T}$, where the terms $\left(a_{41}\;a_{42}\;a_{43}\;a_{44}\;a_{45}\right)$ obtained by calculating the exact and approximate solutions from (7) and (12).

In practical control, the system input is not a continuous signal but is obtained through sampling. We assume that the system input sampling interval is δ, which represents the time interval between adjacent moments. For the length of the FMPC prediction horizon, we have $T_{p}=N\delta$. Where *N* is the prediction horizon. Therefore, within the control step, the sampling time can be represented as $t_{i}=i\delta$, and within the time interval [i,i+1), the ZMP velocity can be denoted as ${\dot{x}}_{z}^{i}$. Furthermore, within the time interval [i,i+1), the ZMP position at any time can be expressed as:

(14) $$\begin{array}{c} x_{z}\left(t\right)=x_{z}^{i}+\left(t-t_{i}\right){\dot{x}}_{z}^{i},\;t\in \left[t_{i},t_{i+1}\right) \end{array}$$

Extending from the current sampling time $t_{k}$ to the control interval $t_{k+N}$, the ZMP velocity vector within the interval can be expressed as:

(15) $$\begin{array}{c} {\dot{X}}_{Z}^{k}={\left({\dot{x}}_{z}^{k}\;\ldots \;{\dot{x}}_{z}^{k+N-1}\right)}^{T} \end{array}$$

This yields the control input variable ${\dot{X}}_{Z}^{k}$ in one MPC control cycle, where the ${\dot{X}}_{Z}^{k}$ denotes the control input variable within a single FMPC prediction horizon.

### 4.2. Multi-Constraint in FMPC

In this part, based on the robot’s motion laws and the characteristics of the flexible C-T model, we construct the multi-constraint integration of FMPC, including motion limit constraints, CoM distance constraints, and boundedness constraints.

#### 4.2.1. Motion Limit Constraints

**Figure 4:**
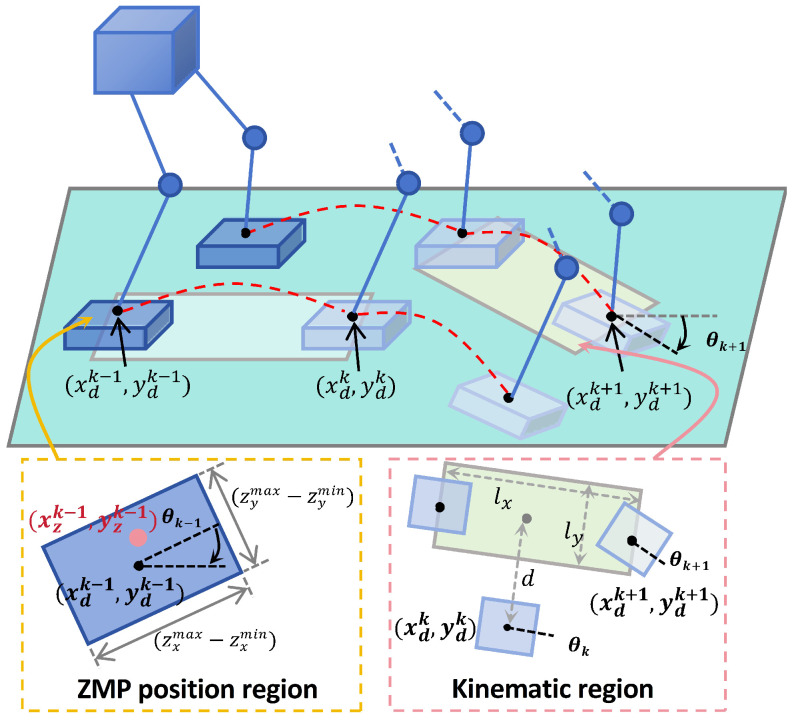
Limit Constraints on Walking State.

During robot walking, maintaining the ZMP within the support polygon of the foot, and at the same time the robot is able to execute the generated ZMP position. Therefore, the Motion Limit Constraints can be divided into ZMP constraints and kinematic constraints, as illustrated in Figure 4, the subscript *d* indicates the desired ZMP position, and θ represents the walking orientation.

As shown in the ZMP position region, the allowable ZMP range is represented by a rectangle with a length of $\left(z_{x}^{\max}-z_{x}^{\min}\right)$ and a width of $\left(z_{y}^{\max}-z_{y}^{\min}\right)$. When the rotation angle θ of the gait is not considered, the following ZMP constraints apply to the current ZMP position $x_{z}^{k-1}$ during the (k−1)th sampling interval:

(16) $$\begin{array}{c} z_{x}^{\min}\leq x_{z}^{k-1}\leq z_{x}^{\max} \end{array}$$

To standardize the expression of various ZMP constraints, we define the ZMP position area as a rectangle with dimensions $d_{x}*d_{y}$. According to (14), at adjacent sampling instants, the next position can be computed based on the previous position and velocity:

(17) $$\begin{array}{cc} x_{z}^{k+1} & =x_{z}^{k}+\delta {\dot{x}}_{z}^{k}\\ x_{z}^{k+2} & =x_{z}^{k}+\delta {\dot{x}}_{z}^{k}+\delta {\dot{x}}_{z}^{k+1}\\ \; & \vdots \\ x_{z}^{k+C} & =x_{z}^{k}+\cdots +\delta {\dot{x}}_{z}^{k+N-1} \end{array}$$

Therefore, at any time (k+i), the following expression can be derived:

(18) $$\begin{array}{c} -\frac{1}{2}d_{x}\leq x_{z}^{k}+\delta \sum_{j=0}^{i-1}{\dot{x}}_{z}^{k+j}-x_{d}^{k+i}\leq \frac{1}{2}d_{x} \end{array}$$

For cases involving a gait with a rotation angle θ, a transformation can be applied using a rotation matrix. Correspondingly, (18) changes to (the same approach can be applied to the y-direction):

(19) $$\begin{array}{c} -\frac{1}{2}d_{x}\leq R_{k+i}^{T}\left(x_{z}^{k}+\delta \sum_{j=0}^{i-1}{\dot{x}}_{z}^{k+j}-x_{d}^{k+i}\right)\leq \frac{1}{2}d_{x} \end{array}$$

When the ZMP constraints are satisfied within the sampling time, they remain valid in continuous time, as $x_{z}\left(t\right)$ is linear during the sampling interval based on (14). Therefore, the constraint is linearly related to the ZMP velocity input. It is worth noting that the constraints are nonlinear with respect to θ, which depends on the direction of the specified gait.

As illustrated in the Kinematic region, the gait at times k+1 and *k* is constrained by the kinematic feasible region:

(20) $$\begin{array}{c} -\frac{1}{2}\left(\begin{array}{c} l_{x}\\ l_{y} \end{array}\right)\leq R_{k}^{T}\left(\begin{array}{c} x_{d}^{k+1}-x_{d}^{k}\\ y_{d}^{k+1}-y_{d}^{k} \end{array}\right)\pm \left(\begin{array}{c} 0\\ d \end{array}\right)\leq \frac{1}{2}\left(\begin{array}{c} l_{x}\\ l_{y} \end{array}\right) \end{array}$$

where ± in the kinematic constraints indicates the different states corresponding to the left and right gaits. the supporting gait is centered at $\left(x_{d}^{k},y_{d}^{k}\right)$ with a direction of $\theta_{k}$. The kinematic feasible region can be defined as a rectangle aligned with $\theta_{k}$ and offset by a certain distance along the y-axis, described as an $l_{s}*l_{y}$ rectangle. The horizontal distance between the left and right gaits is defined as *d*. It is crucial to ensure that the robot’s forward and backward gait remains within its kinematic limits during walking.

Motion limit constraints are formulated by incorporating the ZMP along with kinematic constraint to ensure that the robot’s motion adheres to fundamental kinematic principles.

#### 4.2.2. CoM Distance Constraint

The flexible C-T model extends the conventional C-T model by introducing elastic connections between flexible centers of mass, enabling it to capture more complex dynamic behaviors.

When the distance between the carts’ centers of mass increases, the system’s flexibility increases, improving stability, but the overall dynamic response becomes slower. Conversely, increased system rigidity reduces stability while enhancing the overall dynamic response speed. For ease of converting constraints, we consider the system’s output as the distance between the centers of mass, as follows:

(21) $$\begin{array}{c} y=x_{c_{2}}-x_{c_{1}}=Cx \end{array}$$

Consequently, the constraint on the output y can be obtained. By iterating the system’s state equations, we can convert the constraint on the output y into a constraint on the output ${\dot{x}}_{z}$, as follows:

(22) $$\begin{array}{c} dis_{\min}\leq y\leq dis_{\max} \end{array}$$

CoM distance constraint is imposed to regulate the elastic structure of the flexible C-T model. By constraining the variation in the distance between CoMs, it is possible to control the motion state of $m_{1}$.

#### 4.2.3. Boundedness Constraint

Since the LIP dynamic model contains an unstable subsystem, even if the ZMP is constrained within the foot support polygon, ensuring stable gait characteristics, the CoM trajectories calculated using the LIP model, while satisfying the dynamic equations, may exhibit exponential divergence relative to the ZMP. Moreover, when applying kinematic constraints to the robot, these divergent CoM trajectories become clearly infeasible. The expression for the unstable component can be easily derived from the unstable subsystem of the LIP model:

(23) $$\begin{array}{c} {\dot{x}}_{u}=\eta \left(x_{u}-x_{z}\right) \end{array}$$

As previously discussed, for the unstable solution of the LIP model, we need to find a bounded specific trajectory, ensuring no exponential divergence under current conditions. If the unstable solution $x_{u}$ satisfies specific initial conditions, ensuring that the resulting CoM trajectory is bounded relative to the ZMP, this is called the boundedness condition. The initial condition at time *k* is denoted as $x_{u}\left(t_{k}\right)$, and its analytical solution is:

(24) $$\begin{array}{c} x_{u}\left(t_{k}\right)=x_{u}^{*}\left(t_{k};x_{z}\right)=\eta \int_{t_{k}}^{\infty }e^{-\eta (\tau -t_{k})}x_{z}\left(\tau \right)d\tau \end{array}$$

From the piecewise linear nature of ZMP in (14), we can simplify to obtain:

(25) $$\begin{array}{c} x_{u}\left(t_{k}\right)=\eta \sum_{j=k}^{\infty }\left(e^{-\eta (t_{j}-t_{k})}\int_{0}^{\delta }e^{-\eta s}\left(x_{z}^{j}+s{\dot{x}}_{z}^{j}\right)ds\right) \end{array}$$

where $s=\tau -t_{j}$. The calculation of $x_{u}$ starts at a specific moment, but the FMPC includes a prediction horizon, with the area beyond this called the tail. It is shown that anticipatory tails can predict gait irregularities in both regular and irregular gait sequences, therefore achieving recursive feasibility [36]. Hence, we incorporate anticipatory tails into the boundedness constraint. By processing (25), the boundedness constraint is obtained as:

(26) $$\begin{array}{c} \sum_{i=0}^{N-1}e^{-i\eta \delta }{\dot{x}}_{z}^{k+i}+\sum_{i=N}^{P-1}e^{-i\eta \delta }{\dot{x}}_{z,p}^{k+i}+\sum_{i=P}^{\infty }e^{-i\eta \delta }{\dot{x}}_{z,\infty }^{k+i}=\frac{\eta }{1-e^{-\eta \delta }}\left(x_{u}^{k}-x_{z}^{k}\right) \end{array}$$

where *N* represents the prediction horizon for the FMPC, while *P* indicates the length of the reference ZMP trajectory sequence associated with the specified footstep sequence ${\hat{X}}_{d}^{k}$. ${\dot{x}}_{z,p}$ denotes the ZMP velocity from the control time to the gait time, and ${\dot{x}}_{z,\infty }$ represents the remaining tail after the gait time. Clearly, the boundedness constraint is affected by the system state and the tail state.

### 4.3. Formulation of the QP Problem

In our FMPC framework, every iteration applies the flexible C-T prediction model outlined earlier, together with relevant constraints, to solve the QP problem. Furthermore, the constraints in both the x and y directions are of identical form.

The following vector is defined:

$$\begin{array}{c} {\dot{X}}_{z}^{k}={\left({\dot{x}}_{z}^{k}\;\;\ldots \;\;{\dot{x}}_{z}^{k+N-1}\right)}^{T}\\ {\dot{Y}}_{z}^{k}={\left({\dot{y}}_{z}^{k}\;\;\ldots \;\;{\dot{y}}_{z}^{k+N-1}\right)}^{T} \end{array}$$

At this stage, the QP problem in the FMPC framework can be expressed as:

$$\left\{\;\begin{array}{c} \;\min_{\begin{array}{c} {\dot{X}}_{z}^{k},X_{f}^{k} \end{array}}∥{\dot{X}}_{z}^{k}{∥}^{2}+\omega {∥X_{f}^{k}-X_{d}^{k}∥}^{2}\\ \;\;\;\;\mathrm{subject}\;\mathrm{to}\\ •\;\mathrm{Motion}\;\mathrm{limit}\;\mathrm{constraints}\;(19)\;\mathrm{and}\;(20)\\ •\;\mathrm{CoM}\;\mathrm{distance}\;\mathrm{constraint}\;(22)\\ •\;\mathrm{Boundedness}\;\mathrm{constraint}\;(26)\;\mathrm{for}\;x \end{array}\right.$$

The first term of the cost function depends on all decision variables directly related to the ZMP velocity, while the second term aims to approach the given gait sequence as closely as possible. It is important to note that the solution applies to the *y* direction as well. The motion limit constraints are imposed on both the *x* and *y* directions, whereas the boundedness constraint is separately applied to the *x* and *y* directions. The tail of the Boundedness Constraint can vary depending on the choice, but the same tail should be applied to both the *x* and *y* directions.

**Remark** **3.**  *Within the FMPC framework, motion constraints are applied to limit the fundamental states of robot movement, while boundedness constraints ensure the stability of the CoM trajectory generated by the FMPC approach. Furthermore, we introduce additional center of mass constraints to regulate the model’s inherent flexible characteristics, particularly addressing the spring-damping properties of the flexible C-T model, therefore forming the multi-constraint integration of FMPC.*

### 4.4. FMPC Algorithm

**Figure 5:**
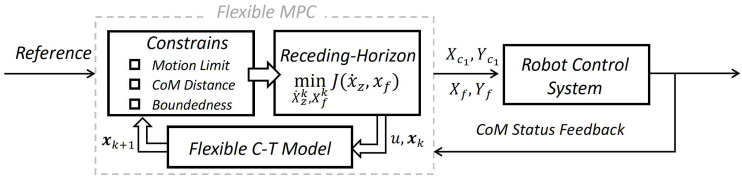
Simplified Block Diagram of FMPC Algorithm.

The simplified FMPC algorithm framework is depicted in Figure 5. Based on the specified reference inputs, constraints are formulated and solved. The flexible C-T model is then used to calculate the CoM state, which guides the robot’s motion. This CoM state is subsequently fed back as the initial condition for the next MPC iteration, achieving closed-loop control.

The complete process of the enhanced FMPC algorithm is outlined in Algorithm 1. The input data consists of the time-series gait steps $\left({\hat{X}}_{d}^{k},{\hat{Y}}_{d}^{k}\right)$, which are used to compute the gait information in the prediction horizon, therefore obtaining the ZMP boundaries over the entire control range, i.e., the upper and lower bounds of the ZMP trajectory. In each iteration, the boundary range for the current prediction horizon is computed. Additionally, as part of the initialization, the starting values of the system control variable, including the positions and velocities of the two CoMs and the initial ZMP position, need to be set.

The flow of the FMPC computation at the $t_{k}$ moment is shown below:

1. By solving the corresponding QP problem, the optimal solution sequences (${\dot{X}}_{z}^{k},{\dot{Y}}_{z}^{k},X_{f}^{k},Y_{f}^{k}$) are obtained.
2. Choose the first control sample from the optimal solution sequences (${\dot{X}}_{z}^{k},{\dot{Y}}_{z}^{k}$) as the system input, and calculate the corresponding values for the next time step based on the current state variables.
3. Transmit the computed footstep positions $\left(X_{f}^{k},Y_{f}^{k}\right)$ and the calculated center of mass positions to the motion planning module, where they are converted into joint angles through kinematic transformation.

Notably, the swing leg trajectory is generated using polynomial fitting based on the previous and next footstep positions, so in each iteration, only the first footstep in the gait sequence needs to be sent to the motion planning module.
**Algorithm 1** Flexible MPC Method**Input:**Initial state $x_{0}$Reference gait sequence ${\hat{X}}_{d}^{k}$Prediction horizon *N*Gait duration *D*Dynamic model f(x,u)(13)**Output:** Optimal control sequence $x_{c_{1}}$.1:Initialize iteration counter k←02:**while** k<D **do**3:   Solve the quadratic programming problem for the control input sequence $u_{t:t+N-1}$:
$$\min_{\begin{array}{c} {\dot{X}}_{z}^{k},X_{f}^{k} \end{array}}∥{\dot{X}}_{z}^{k}{∥}^{2}+\omega {∥X_{f}^{k}-X_{d}^{k}∥}^{2}$$   subject to: constrains (19), (20), (22) and (26)4:   Apply the first control input $u_{t}^{*}$ from the optimized sequence5:   Predict future states: $x_{k+1}=f\left(x_{k},u_{k}\right)$6:   Update system state $x_{k+1}$ and increment k←k+17:**end while**

## 5. Experiments

In this section, we validate the boundedness and feasibility of ZMP trajectory prediction using the flexible C-T model within the FMPC framework through simulations and comparative experiments, demonstrating the method’s effectiveness.

### 5.1. Experimental Setup

In our simulations, we conducted preliminary simulations based on the flexible C-T model to emphasize the main characteristics of our approach. For robot gait control, we then compare the performance of the proposed Flexible MPC (FMPC) framework with Standard MPC [23] and Intrinsically Stable MPC (ISMPC) [35]. While ISMPC relies on the extended dynamics of the LIP model with ZMP velocity as the control variable, it does not account for the robot’s internal flexibility. The standard MPC method, using CoM jerk as a control variable as in [9], also lacks bounded constraints. Both methods, however, apply the same ZMP constraint.

We performed simulation experiments in MATLAB R2023a to assess the proposed method. We set various sampling times δ and a prediction horizon *N* for each MPC iteration and provided a series of fixed step lengths for the gait (e.g., single support time of 0.15 s and double support time of 0.35 s). The permissible ZMP landing area is a 0.05m×0.05m rectangular region, marked with a red box. The generated CoM trajectory is shown by a yellow curve, and the ZMP trajectory is shown by a blue curve. Under stable conditions, the CoM and ZMP trajectories maintain symmetry and stability, with the green curve representing the ZMP trajectory for the next prediction horizon.

For simplicity, we assumed the footstep sequence is fixed, meaning that in the QP problem, ω in the loss function tends toward infinity. This implies that the provided footstep sequence satisfies the kinematic constraints (20). The QP problem was solved using the quadprog function.

### 5.2. Boundedness Comparison Experiment

Simulation experiments were conducted under different conditions, with the range of a predefined footprint sequence serving as the reference ZMP range. The stability of the proposed method was validated by varying the prediction horizon *N*, while its adaptability was tested by adjusting the sampling time δ.

#### 5.2.1. Performance Verification Under δ=0.01 s, *N* Decreasing

**Figure 6:**
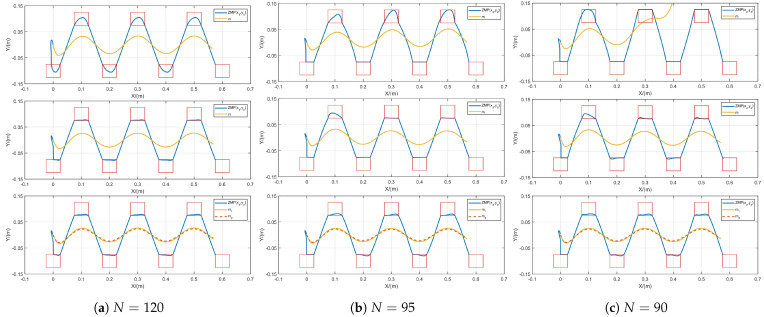
Gait generation for different prediction horizons for standard MPC [23] (**top**), ISMPC [35] (**middle**) and FMPC (**bottom**) at δ=0.01 s.

To verify the stability of the CoM trajectories generated by FMPC under different prediction horizons, we test them given the sampling time δ=0.01 s, decreasing *N* sequentially. We set the CoM height to h=0.6 m for each model, and for the flexible C-T model, $m_{1}$ was set to 4 kg and *k* to 1000. Additionally, $m_{2}$ and *b* were adjustable to influence the system. In the simulation, we assigned $m_{2}=1$ kg and b=2 and observed the variations among the three methods by adjusting the prediction horizon *N*, as illustrated in Figure 6. When the prediction horizon is N=120, which corresponds to $T_{c}=1.2$ s, the simulation depicted in Figure 6a indicates that all three MPC methods can produce stable CoM trajectories. From the perspective of ZMP trajectories, the ISMPC and our FMPC methods were quite similar, as both control ZMP variation directly in the loss function, resulting in smoother ZMP trajectories.

Next, we reduced the prediction horizon to N=95, or $T_{c}=0.95$ s. The simulation, shown in Figure 6b, demonstrates that while all three methods still produce stable CoM trajectories, the standard MPC’s CoM and ZMP trajectories show an asymmetric trend. It is observed that the LIP model’s movement tends to tilt upward, leading to inconsistent tilt directions of the left and right support feet. ISMPC has this tendency on the first step, which is recovered later by active constraints. Our FMPC does not have this problem.

We further reduced the prediction horizon to $T_{c}=0.9$ s. The simulation results are illustrated in Figure 6c. The CoM trajectory generated by the standard MPC has begun to diverge due to the short prediction horizon, which prevents stabilization of the CoM trajectory through jerk minimization. While both ISMPC and our approach maintain stable CoM trajectories, the first step of ISMPC still exhibits an asymmetrical state.

**Figure 7:**
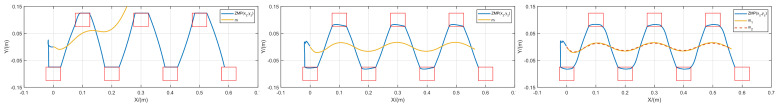
Gait generation for standard MPC [23] (**left**), ISMPC [35] (**middle**), and FMPC (**right**) at h=1.2 m.

Furthermore, we discovered an intriguing phenomenon: when CoM height is increased to h=1.2 m while maintaining a stable prediction horizon of N=120, the standard MPC quickly diverges, whereas both ISMPC and our FMPC maintain boundedness in the CoM. The increase in center of mass height effectively reduces gravitational acceleration *g*, resembling gait generation in a weightless environment (Figure 7). Therefore, we can conclude that merely augmenting the ZMP position constraints does not fully avert instability.

Therefore, under a certain sampling time, as the prediction horizon gradually decreases, the CoM trajectory generated by standard MPC tends to diverge, and the CoM trajectory produced by ISMPC becomes asymmetric, while the CoM trajectories generated by the FMPC remain symmetric and stable, which can be seen that the FMPC still maintains good stability when coping with the decrease of the prediction horizon.

#### 5.2.2. Performance Verification Under δ=0.008 s, *N* Decreasing

**Figure 8:**
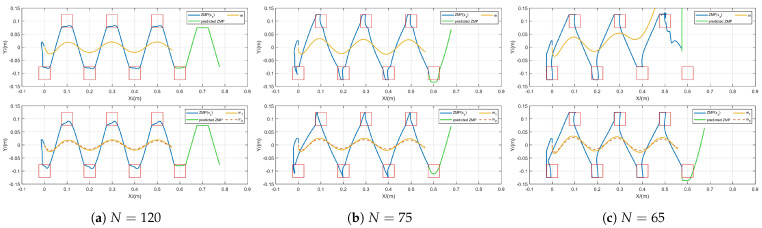
Gait generation for different prediction horizons for ISMPC [35] (**top**) and FMPC (**bottom**) at δ=0.008 s.

To verify that the FMPC can adapt to different sampling times δ, we decrease the sampling time when δ=0.008 s, the system frequency will increase from 100 Hz to 125 Hz, and we use Equation (17) to calculate the predicted ZMP trajectory for the next prediction horizon. It can be assumed that a signal interruption occurs during the robot’s motion in order to allow the robot a buffer interval to execute ZMP trajectories within the predicted range up to a stable stopping state. The previous experimental results have already shown that standard MPC fails to meet various control demands, so we will now compare it with the ISMPC method. We conducted simulations with different prediction horizons, and the results are shown in Figure 8. First, we set the prediction horizon to N=120, as illustrated in Figure 8a. Both methods successfully generate stable CoM trajectories, and the predicted ZMP trajectory for the upcoming prediction horizon closely matches the actual ZMP trajectory while also adhering to the ZMP constraints.

When we decrease the prediction horizon to N=75 (Figure 8b), we observe that both methods’ ZMP trajectories show a sharper trend. With the reduced sampling time and shorter prediction horizon, the system’s dynamic response becomes more frequent, and the controller’s computation speed increases, possibly causing more dramatic input-output variations, leading to sharper ZMP trajectories. Furthermore, we also notice that part of the ZMP trajectory predicted by ISMPC has exceeded the ZMP constraint region, while ours has not, reflecting the flexible nature of our FMPC.

As we continue to reduce the prediction horizon to N=65 (Figure 8c), it becomes evident that the ISMPC shows signs of divergence. This is likely due to the accumulation of discretization errors in the system model with the shortened sampling time, making it harder to accurately capture the system’s dynamic characteristics. In contrast, our FMPC framework incorporates an additional flexibility layer (spring-damper model and second CoM) in the prediction model, which helps absorb the shocks caused by rapid changes, providing extra buffering capacity and maintaining system stability.

Simulation results demonstrate that the bounded constraints we introduced ensure that the CoM trajectory remains bounded relative to the ZMP trajectory. Through a comparative analysis with standard MPC and ISMPC, we found that, with the same sampling time, reducing the prediction horizon of MPC causes the CoM trajectory generated by standard MPC to diverge while ISMPC and FMPC remain stable. When the sampling time is reduced and the MPC control period is shortened, the CoM trajectory generated by ISMPC also diverges, but FMPC remains stable and bounded. Additionally, in the case of signal interruptions, the predicted ZMP trajectory in FMPC stays within the constraints, demonstrating FMPC’s stability under different conditions.

### 5.3. Parametric Sensitivity Analysis of the Flexible C-T Model

The previous experiments indicate that the FMPC framework, compared to ISMPC and standard MPC, has better flexibility, enabling it to adapt to high-frequency, low-prediction-horizon-environment changes. Given that the flexible C-T model adds extra elastic damping and a second CoM compared to the simplified LIP model, we adjust these parameters to evaluate their different performance characteristics. Based on the above experimental results, it is demonstrated that the FMPC framework exhibits higher flexibility in high frequency and low prediction horizon compared to ISMPC and standard MPC.

**Figure 9:**
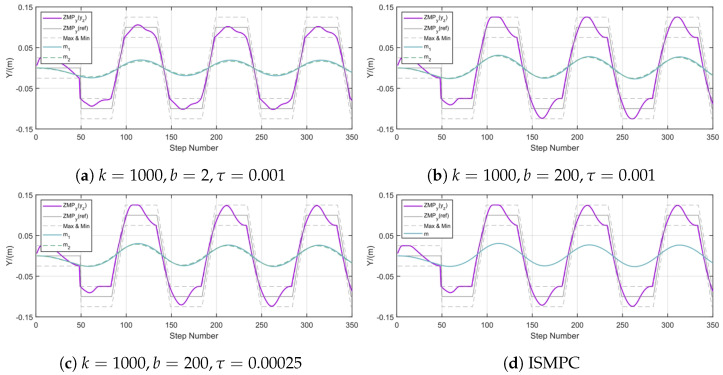
Comparison of different choices of *k*, *b* and τ and ISMPC.

Figure 9 presents the ZMP variation results along the y-axis when the parameters change under conditions of h=0.9 m, N=80, and δ=0.01 s, and the ZMP tracking results from ISMPC. It is observed that with a lower *b* value, the ZMP tracking error is minimal. As the *b* value increases, adjustments to τ are needed to compensate for the resulting tracking error. From Figure 9, we can see that under the conditions of Figure 9a, the ZMP trajectory is tracked more effectively, achieving better results compared to the ISMPC ZMP tracking trajectory shown in Figure 9d. Therefore, when tracking the ZMP trajectory, our FMPC framework can adjust the ZMP trajectory by fine-tuning the related parameters without incorporating ZMP tracking into the cost function like standard MPC, which not only increases computational load but also directly impacts the optimal solution.

**Figure 10:**
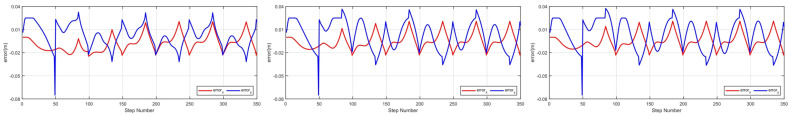
Different second CoM mass share:20%, 33.3% and 42.8% of the total mass.

We can also fine-tune the mass of the second CoM ($m_{2}$). As shown in Figure 10, by setting different masses for the second center of mass, we observed the error between the generated ZMP trajectory and the reference ZMP trajectory. It becomes apparent that as the mass increases, the ZMP error grows, which is particularly evident from the third curve in Figure 10.

**Figure 11:**
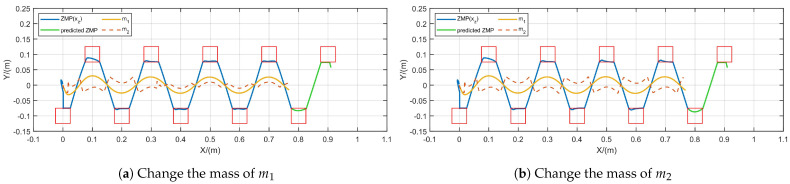
The effect of changes in CoM mass on gait generation.

In addition, we performed tests to evaluate CoM mass variation during gait generation. We initialized $m_{1}=40$ kg and $m_{2}=1$ kg under conditions of h=0.6 m, N=90, δ=0.01 s, with a step length of 0.1m for 8 steps. As depicted in Figure 11, we varied the masses of $m_{1}$ and $m_{2}$ during gait generation. In Figure 11a, $m_{1}$ was reduced to 20 kg at the fourth step while $m_{2}$ remained unchanged, resulting in a reduced motion of $m_{2}$. This reduction occurred as the decrease in $m_{1}$’s mass minimized its influence on $m_{2}$, while $m_{1}$ maintained stable and bounded motion. In Figure 11b, $m_{2}$’s mass increased to 10 kg at the fourth step, with $m_{1}$ unchanged. $m_{2}$’s motion remained largely unaffected since it primarily reflects joint flexibility and swing leg disturbances, which minimally affect the overall CoM $m_{1}$. These findings indicate that the CoM/ZMP trajectories produced by the FMPC approach remain stable and bounded despite CoM mass variations.

It is worth noting that reducing the mass does not always guarantee a more accurate ZMP trajectory. Due to the inherent characteristics of the flexible C-T model, when $m_{2}$ reaches a critical value, the MPC might fail to solve. Choosing an optimal mass for the second center of mass not only helps simulate internal robot collisions but also provides flexibility when both feet are in contact with the ground.

### 5.4. Irregular Gait Generation Expansion

To broaden the applicability of our MPC for various stepping scenarios, we consider gait generation under multiple conditions: speed change, direction change, and lateral force. For simplicity, we assume no change in foot orientation, i.e., $\theta =0^{\circ }$. In the MPC implementation, we select a sampling time of δ=0.01 s, a single support phase of 0.15 s, a double support phase of 0.35 s, and a ZMP allowable landing area of a 0.05m×0.05m rectangular zone.

#### 5.4.1. Gait Generation with Variable Speed

**Figure 12:**
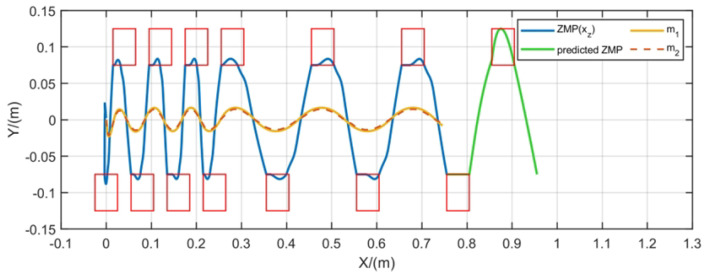
Gait generation with variable velocity.

**Figure 13:**
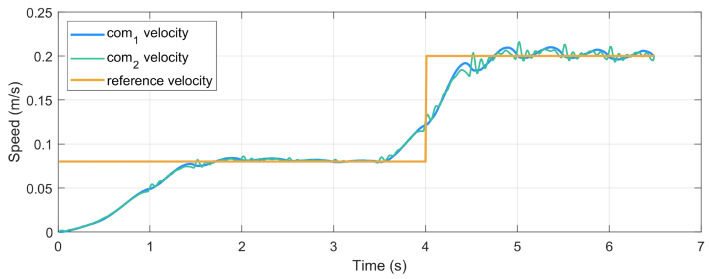
CoM speed tracking during walking.

To test the ability of FMPC to handle changes in walking speed during gait generation, we perform steps of varying lengths, enabling the robot to cover different stride distances within the same timeframe. Because the duration of all steps remains consistent, this allows for the simulation of variable-speed walking. The simulation results are displayed in Figure 12, demonstrating stable CoM and ZMP trajectories. Additionally, the predicted ZMP trajectory in the future prediction interval remains within the constraint region. Figure 13 illustrates the velocity tracking during the motion process. It is evident that, due to dynamic model errors, the measured CoM velocity shows slight variations compared to the desired velocity, yet the overall tracking direction remains aligned.

#### 5.4.2. Gait Generation with Change of Direction

**Figure 14:**
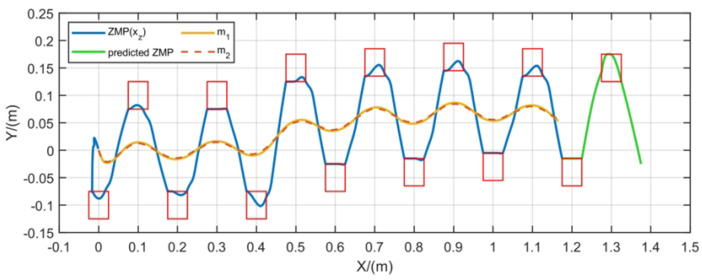
Gait generation with change of direction.

To test the FMPC method’s ability to handle changes in walking direction during gait generation, we performed gait tests with varying directions, maintaining a constant CoM speed. During the walking process, the direction of the CoM changes, as illustrated in Figure 14. The asymmetry observed in the ZMP and CoM trajectories results from the footsteps not aligning with the path during that phase; however, both the ZMP and CoM trajectories remain stable and bounded.

#### 5.4.3. Gait Generation with Lateral Force

**Figure 15:**
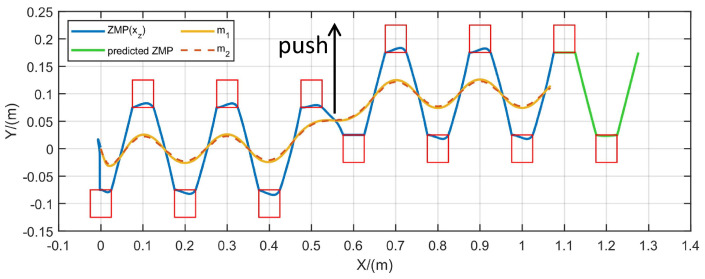
Gait generation with lateral force.

To evaluate the FMPC’s robustness to sudden external disturbances during gait generation, a step lateral force was applied while maintaining a constant CoM velocity. As shown in Figure 15, the lateral step force was applied at the sixth step. The CoM and ZMP trajectories generated by FMPC remained symmetric and bounded throughout, and the predicted ZMP trajectory stayed within limits, unaffected by the lateral force.

### 5.5. Robots Simulation: A Replication of Real-World Applications

**Figure 16:**
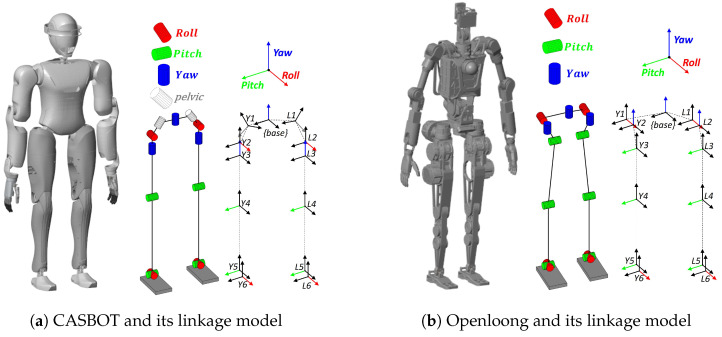
Robot joint modelling in MATLAB.

To validate the effectiveness of the proposed method, we chose the bipedal humanoid robots CASBOT and Openloong as test platforms. The simulation demonstrations based on CASBOT and Openloong effectively replicate real-world walking scenarios. CASBOT’s CoM is at a height of 0.93 m, and Openloong’s CoM is 0.956 m. Both have 12 degrees of freedom in their legs, and we build their simulation models in MATLAB as shown in Figure 16, with the ankle joint providing pitch and roll degrees of freedom and the knee joint providing pitch degrees of freedom. For the flexible C-T model, we assume that the weight of CoM $m_{1}$ is 40 kg, and the weight of the second CoM $m_{2}$ is 10 kg. In addition, we use polynomial interpolation to simulate the motion of the swing leg.

**Figure 17:**
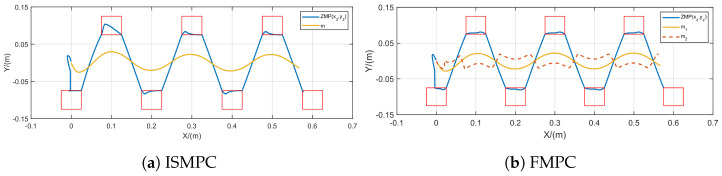
Gait generation for ISMPC [35] and FMPC at h=0.8 m.

Initially, we compare the proposed method with ISMPC under the conditions δ=0.01 s and N=100. When the robot walks, CoM needs to be appropriately lowered, so let h=0.8 m, with knee bending as the starting state. Figure 17 demonstrates that both methods show stable trajectories; however, the CoM trajectory generated by ISMPC has a minor deviation in the first step, while our FMPC consistently maintains symmetry.

**Figure 18:**
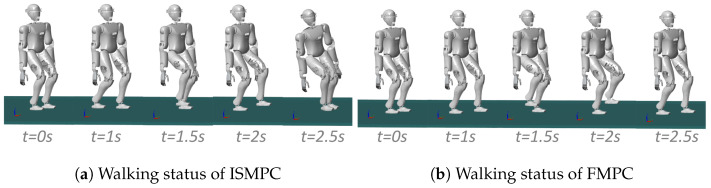
CASBOT: Comparison of ISMPC [35] and FMPC walking status.

**Figure 19:**
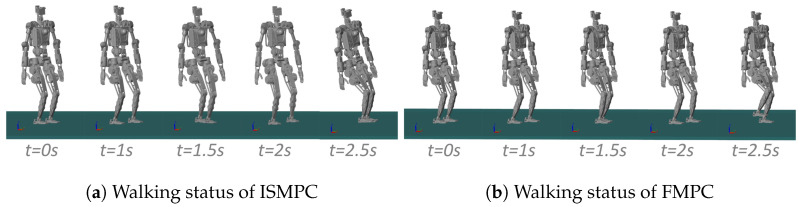
Openloong: Comparison of ISMPC [35] and FMPC walking status.

We set up the simulation environment in MATLAB’s Simulink, set the contact state between the feet and the ground as the four rectangles of the foot rectangle, and set robots to float, increasing the gravitational acceleration g=9.8 m/$s^{2}$. The joint friction and ground friction of the robot are set by default. Utilizing the CoM trajectory generated by FMPC and interpolating between footstep positions, we determined the CoM and foot-end positions for each motion instant. Joint angles were then calculated via kinematic analysis based on the robot’s link model and applied to CASBOT and Openloong, whose walking processes are shown in Figure 18 and Figure 19. From the walking process of Figure 18a as well as Figure 19a, we can see that the robot rolls over when walking up to 2.5 s, whereas in Figure 18b as well as Figure 19b, we can see that when using our method. The robot still maintains a stable walk.

**Figure 20:**
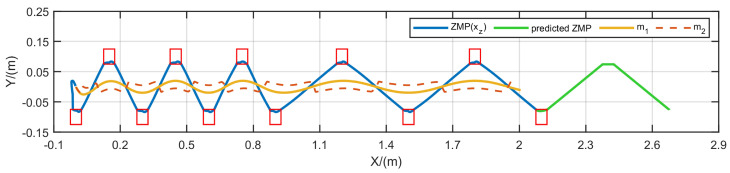
Variable velocity walking trajectory at h=0.8 m.

A variable-speed walking process was planned for the robot, as depicted in Figure 20. In the first phase, the speed is set to 0.15 m/s, and in the second phase, it is increased to 0.3 m/s. The CoM/ZMP trajectories remain stable and bounded, and the predicted ZMP trajectory also stays within a stable region.

**Figure 21:**
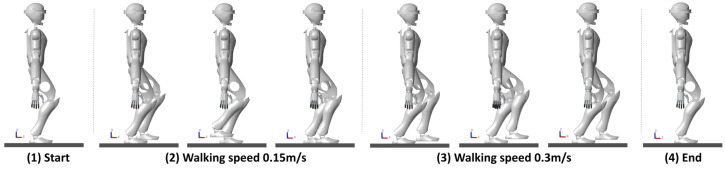
CASBOT: Simulated walking at different speeds.

**Figure 22:**
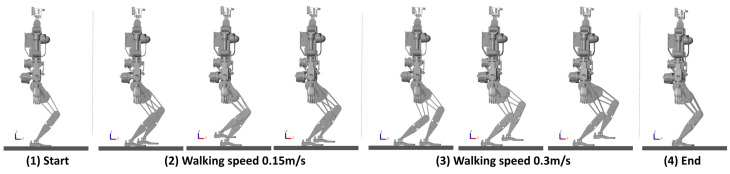
Openloong: Simulated walking at different speeds.

The walking state of the robots is illustrated in Figure 21 and Figure 22: beginning from rest, the robot adopts a semi-squat position; in the first phase, the walking speed is set to 0.15 m/s; when a command to change speed is received, the robot’s walking speed increases to 0.3 m/s in the second phase; upon finishing the walk, the robot resumes a stable semi-squat posture.

**Figure 23:**
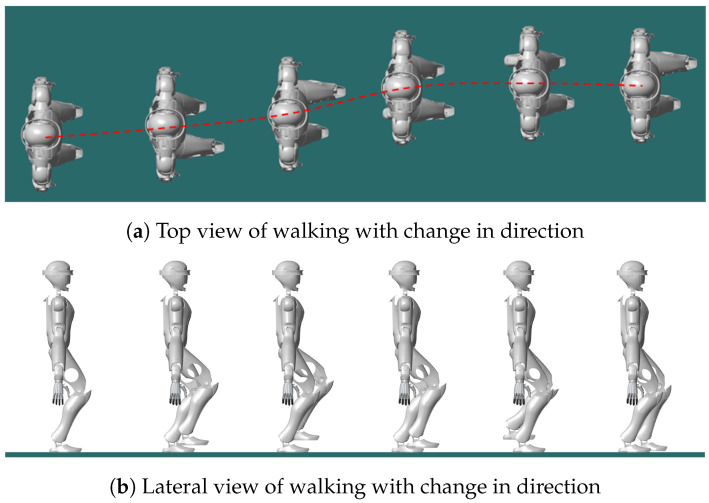
CASBOT: Simulated walking with a change in direction.

To assess the FMPC’s capability in generating 2D gaits, we tested the trajectory shown in Figure 14 on the CASBOT robot. As shown in Figure 23, the robot starts in a semi-squat posture. During walking, its trajectory shifts gradually to the left, corresponding to a directional change in gait. Throughout the transition between walking states, the robot maintained stability, confirming the applicability of the 2D gait trajectories generated by FMPC for robot walking.

We confirmed the FMPC’s ability to generate bounded gaits via comparative analyses of different sampling times and prediction horizons. Furthermore, ZMP trajectory tracking tests indicated that tuning FMPC parameters enhances ZMP tracking performance. Additional gait experiments and tests on the CASBOT and Openloong robots revealed the FMPC method’s excellent flexibility and stability.

## 6. Conclusions

This paper proposes an FMPC framework that integrates joint dynamics through an enhanced flexible C-T model with an elastic damping structure and an additional CoM while incorporating multiple constraints to address the inability of traditional MPC methods based on LIP or C-T model to approximate the intrinsic properties of humanoid robots. The FMPC framework enables the generation of stable walking trajectories for humanoid robots under various conditions. Simulation comparisons highlight the superior stability and adaptability of FMPC under high-frequency, low-prediction-horizon scenarios, as well as its capability to handle variable-speed and variable-direction gait generation. Additionally, experimental simulations on the CASBOT and Openloong robots validate the effectiveness of the proposed method. This approach demonstrates significant potential for enhancing humanoid robot stability and adaptability in complex environments, laying a solid foundation for practical deployment. Future research will focus on further strengthening the MPC framework and extending it to flexible 3D gait generation, with consideration of continuous disturbances.

## Data Availability

Data are contained within the article.
