# Correction: Yang et al. Flexible Model Predictive Control for Bounded Gait Generation in Humanoid Robots. *Biomimetics* 2025, *10*, 30

**DOI:** 10.3390/biomimetics10080540

**Published:** 2025-08-18

**Authors:** Tianbo Yang, Yuchuang Tong, Zhengtao Zhang

**Affiliations:** 1 Institute of Automation, Chinese Academy of Sciences, Beijing 100089, China; yangtianbo2022@ia.ac.cn (T.Y.); yuchuang.tong@ia.ac.cn (Y.T.); 2 The School of Artificial Intelligence, University of Chinese Academy of Sciences, Beijing 100049, China

## Additional Affiliation(s)

In the published publication [1], there was an error regarding the affiliation(s) for Tianbo Yang, Yuchuang Tong, Zhengtao Zhang. In addition to affiliation (1), the updated affiliation(s) should include as follows: 2. The School of Artificial Intelligence, University of Chinese Academy of Sciences, Beijing 100049, China. The authors state that the scientific conclusions are unaffected. This correction was approved by the Academic Editor. The original publication has also been updated.
